# Antimicrobial Sensitivity in Enterobacteria from AIDS Patients, Zambia

**DOI:** 10.3201/eid0801.010018

**Published:** 2002-01

**Authors:** James Mwansa, Kabanga Mutela, Isaac Zulu, Beatrice Amadi, Paul Kelly

**Affiliations:** * University of Zambia School of Medicine, University Teaching Hospital, Lusaka, Zambia; † St. Bartholomew’s & Royal London School of Medicine, London, United Kingdom

**Keywords:** antimicrobial resistance, enterobacteria, septicemia, diarrhea, Africa, HIV infection

## Abstract

Enterobacteria contribute to two serious clinical syndromes seen in African AIDS patients: diarrhea and septicemia. In West Africa, prophylaxis with sulfamethoxazole-trimethoprim (SXT) reduced illnesses. We report reduced sensitivity of enterobacteria to available antimicrobial agents in Zambia, with only 22% of nontyphoidal salmonellae and 6% of shigellae sensitive to SXT.

Diarrhea and septicemia, two of the most important clinical problems of African AIDS patients, are both associated with high rates of illness and death. Treatment with antimicrobial agents may play an important role in reducing illness and possibly death. Chemoprophylaxis (for example, with cotrimoxazole or sulfamethoxazole-trimethoprim [SXT]) has been shown to be effective in reducing illness and death (*1*,*2*). The evidence base relating to patterns of antimicrobial resistance in Africa is small, and antimicrobial agents are often chosen on the basis of availability and expense. Some evidence indicates that resistance patterns vary across Africa, with resistance to SXT in nontyphoidal salmonellae of 14% in Abidjan and 83% in Malawi (*3*). We report the prevalence of infection with three major enterobacteria in Zambian adults and children with AIDS, followed by an analysis of antimicrobial sensitivity patterns.

## The Study

To determine the prevalence of infection in adults and children, cultures were performed by standard techniques on fecal samples from several groups of adults and children, all residents of Lusaka, the capital of Zambia. Group A was 124 adults and 105 children, all HIV-seropositive patients with persistent diarrhea, studied from 1995 to 1999 during three clinical trials of antiprotozoal or nutritional therapies. The average number of samples tested was 2.1 for each adult and 2.8 for each child. Group B was 216 adults enrolled in a longitudinal study of intestinal infection in a cohort of adults in a representative urban community in Lusaka; this group was studied to provide an estimate of asymptomatic carriage rates.

To define the profile of antimicrobial sensitivity, isolates from Group A were analyzed together with additional isolates of nontyphoidal salmonellae, *Shigella dysenteriae*, and *S. flexneri* from a third source (Group C): routine stool or blood cultures from AIDS patients in the University Teaching Hospital from 1995 to 1999. All isolates were cultured and tested for antimicrobial susceptibility by using standard antimicrobial discs (Oxoid Ltd, Basingstoke, UK) on Mueller-Hinton agar. Zones of growth inhibition were compared with standard tables (*4*), and control organisms of known sensitivity were tested beside clinical isolates for verification.

Of 124 adults with persistent diarrhea in Group A, 6 (5%) were infected with nontyphoidal *Salmonella* spp. and 9 (7%) with *S. flexneri* or *S. dysenteriae*. Of 105 children with persistent diarrhea, also in Group A, 21 (20%) were infected with nontyphoidal *Salmonella* spp. and 3 (3%) with *S. flexneri* or *S. dysenteriae*. In Group B, 7 (4%) of 174 adults had one or more fecal samples positive for nontyphoidal *Salmonella* spp. in one year (1999), and 10 (6%) had one or more positive for *S. flexneri* or *S. dysenteriae*. As each adult submitted samples approximately monthly (for a total of 1,440 samples), the point prevalence in these asymptomatic adults was <1% for either infection.

Studies of patients with HIV-related persistent diarrhea in other countries in Africa have found the prevalence of enterobacterial infection to be higher. In Rwanda and Kenya, prevalences of nontyphoidal salmonellae were 11% and 16%, respectively, and of shigellae were 22% and 4%, respectively (*5*,*6*). In recent years, HIV seroprevalence in Lusaka has been estimated to be 22% to 30% (*7*), and the overall rate of HIV-related diarrhea is high (*8*).

## Conclusions

Antimicrobial sensitivity patterns indicate that resistance is a substantial problem among enterobacteria in Lusaka (Table). The isolates we tested came from a tertiary hospital, which may have resulted in some selection bias, as treatment failures may be overrepresented in such patients. However, only a few bacteria tested were sensitive to SXT, in marked contrast to data from West Africa and more closely resembling the situation in Malawi (*3*). This level of resistance may compromise the usefulness of SXT in preventing bacterial infection in HIV-infected persons, although any effect in preventing *Pneumocystis carinii* pneumonia or isosporiasis would be valuable. Emergence of resistance to SXT was noted in San Francisco after its widespread use as prophylaxis against pneumocystosis (*9*). For treatment of infection with these enterobacteria in Zambia, only the more expensive antimicrobial agents now seem to be reliable. Providing effective, affordable parenteral antimicrobial agents for the efficient treatment of septicemic infection in hospitals and health centers is likely to be difficult. As clinical response sometimes occurs even when susceptibility testing in vitro suggests that the antimicrobial agent used is ineffective, controlled clinical trials are needed for these infections in different geographic regions of Africa.

**Table:** Summary of antimicrobial sensitivity patterns for three enterobacteria isolated from patients with HIV-related persistent diarrhea in Zambia

Antimicrobial agent^a^	No. sensitive (%)		
	Nontyphoidal salmonellae	*Shigella flexneri*	*S. dysenteriae*
Tetracycline	37 (6)	2 (6)	3 (16)
Chloramphenicol	36 (77)	7 (22)	8 (48)
Gentamicin	119 (75)	24 (77)	18 (95)
Sulphamethoxazole-trimethoprim	25 (22)	3 (10)	0 (0)
Amoxycillin	74 (48)	9 (30)	7 (37)
Amoxycillin-clavulanic acid	95 (60)	27 (87)	12 (63)
Cephalexin	105 (66)	23 (74)	17 (89)
Cefuroxime	93 (59)	11 (35)	16 (74)
Cefotaxime	149 (88)	28 (90)	19 (95)
Nalidixic acid	107 (67)	31 (100)	19 (100)
Ciprofloxacin	157 (99)	30 (97)	18 (95)
Erythromycin	22 (14)	0 (0)	4 (21)
Azithromycin	64 (92)	9 (100)	19 (100)

^a^158 isolates of nontyphoidal salmonellae, 31 isolates of *S. flexneri*, and 19 isolates of *S. dysenteriae* were tested against all these antimicrobial agents except for azithromycin, against which 69, 9, and 19 isolates were tested respectively.

Antimicrobial sensitivity appeared to decrease from 1995 to 1999, when these isolates were being collected. For example, over this period gentamicin resistance increased from 0% to 32% in *S. flexneri* and from 0% to 34% in nontyphoidal salmonellae (p<0.001). In *S. flexneri*, cefuroxime resistance increased from 22% to 88% and cephalexin resistance from 18% to 42% over the same period (p = 0.001). The scale of use of cephalosporins in the community did not suggest that selection pressure for resistance was likely to be high. Mechanisms of resistance to cephalosporins include reduced permeability and modification of penicillin-binding protein, and emergence appears to be rapid.

Which antimicrobial agents could be recommended for treatment of bacteremic nontyphoidal salmonellosis? The most reliable results are likely to be obtained with fluoroquinolones or azithromycin, but these are expensive and their availability is limited. Gentamicin or chloramphenicol are less expensive and would be acceptable alternatives, although the probability of adverse effects is greater. Unfortunately, few antimicrobial compounds that are likely to be effective and affordable could also be given easily and safely to AIDS patients in primary and secondary care settings in Zambia. As the epidemic in Zambia enters its third decade, the situation is likely to worsen if no action is taken. We have been able to reduce antimicrobial resistance in *Vibrio cholerae* by instituting a policy of rotating the recommended antimicrobial agents during epidemics, thereby prolonging the useful life of affordable antimicrobial agents (J. Mwansa, unpub. obs.). This strategy could be extended to enterobacterial infection in AIDS. We are also considering clinical trials with combinations of antimicrobial drugs to treat these clinical syndromes in AIDS patients. As antimicrobial sensitivity patterns seem to vary across Africa, it may be difficult to generalize the results of clinical trials from one part of the continent to another.
