# Antimicrobial Sensitivity in Enterobacteria from AIDS Patients, Zambia

**DOI:** 10.3201/eid0805.020501

**Published:** 2002-05

## Correction, Vol. 8, No. 1

In the article “Antimicrobial Sensitivity in Enterobacteria from AIDS Patients, Zambia,” by James Mwansa et al., errors were made in calculations for the table on page 93. The corrected table appears below and online at http://www.cdc.gov/ncidod/eid/vol8no1/01-0018.htm. We regret any confusion these errors may have caused.

**Table:** Antibiotic sensitivity patterns for three enterobacteria isolated from patients with HIV-related persistent diarrhea in Zambia

Antimicrobial agent^a^	Nontyphoidal salmonellae	*Shigella flexneri*	*Shigella dysenteriae*
	No. sensitive (%)	No. sensitive (%)	No. sensitive (%)
Tetracycline	37 (23)	2 (6)	3 (16)
Chloramphenicol	36 (23)	7 (23)	8 (42)
Gentamicin	119 (75)	24 (77)	18 (95)
Sulphamethoxazole-trimethoprim	25 (16)	3 (10)	0 (0)
Amoxycillin	74 (47)	9 (29)	7 (37)
Amoxycillin-clavulanic acid	95 (60)	27 (87)	12 (63)
Cephalexin	105 (66)	23 (74)	17 (89)
Cefuroxime	93 (59)	11 (35)	16 (84)
Cefotaxime	149 (94)	28 (90)	19 (100)
Nalidixic acid	107 (68)	31 (100)	19 (100)
Ciprofloxacin	157 (99)	30 (97)	18 (95)
Erythromycin	22 (14)	0 (0)	4 (21)
Azithromycin	64 (93)	9 (100)	19 (100)

^a^One hundred fifty-eight isolates of nontyphoidal salmonellae, 31 of *S. flexneri*, and 19 of *S. dysenteriae* were tested against all these antimicrobial agents, except for azithromycin, against which 69, 9 and 19 isolates were tested, respectively.

